# Standardized* Kaempferia parviflora* Wall. ex Baker (Zingiberaceae) Extract Inhibits Fat Accumulation and Muscle Atrophy in* ob/ob* Mice

**DOI:** 10.1155/2018/8161042

**Published:** 2018-05-28

**Authors:** Sunkyu Lee, Changhee Kim, Dowan Kwon, Mi-Bo Kim, Jae-Kwan Hwang

**Affiliations:** Department of Biotechnology, College of Life Science and Biotechnology, Yonsei University, Seoul 03722, Republic of Korea

## Abstract

Obesity, a metabolic disorder caused by an imbalance between energy intake and energy expenditure, is accompanied with fat accumulation and skeletal muscle atrophy.* Kaempferia parviflora* Wall. ex Baker, also called black ginger, is known to increase physical fitness performance and improve energy metabolism. In this study, we investigated whether* Kaempferia parviflora* extract (KPE) alleviates both obesity and muscle atrophy using* ob/ob* mice. Wild-type C57BL/6J and* ob/ob* mice were provided with a normal diet ad libitum, and* ob/ob* mice were orally given KPE at a dose of 100 mg/kg/day or 200 mg/kg/day for eight weeks. KPE significantly decreased body weight, fat volume, and fat weight without affecting appetite. It inhibited the expression of adipogenic transcription factors and lipogenic enzymes by upregulating AMP-activated protein kinase (AMPK) in epididymal fat. In contrast, it markedly increased the muscle fiber size, muscle volume, and muscle mass, resulting in the enhancement of muscle function, such as exercise endurance and grip strength. On the molecular level, it activated the phosphatidylinositol 3 kinase (PI3K)/Akt pathway, a key regulator in protein synthesis in skeletal muscle. KPE could be a promising material to alleviate obesity by inhibiting adipogenesis, lipogenesis, and muscle atrophy.

## 1. Introduction

Obesity is a chronic metabolic disorder caused by an imbalance between energy intake and energy expenditure. When excessive energy intake is prolonged, the excess energy develops into fat accumulation [1, 2]. Abnormal fat growth results in various metabolic diseases including hyperlipidemia, hypertension, and type 2 diabetes [3, 4]. The reason why obesity occurs with various metabolic diseases is that our body can store fat without limitations. When the capacity of adipose tissue to store triglycerides exceeds, extra lipids that cannot be accumulated in the adipose tissue much longer infiltrate into peripheral organs such as the liver, heart, and skeletal muscle and cause dysfunction of the organs [1, 5]. Therefore, decreasing fat accumulation by regulating energy metabolism is a key strategy for treating obesity and obesity-related metabolic disorders [6, 7].

Recent studies have found a reduced quantity and quality of skeletal muscle in obesity [8, 9]. Genetic mice models of obesity that were leptin- or leptin receptor-deficient showed lower muscle mass than their wild-type counterparts [10]. Additionally, a quantitative and qualitative reduction of skeletal muscle and a decrease in physical endurance were observed in obese people [11, 12]. Because skeletal muscle plays such critical roles in exercise, energy expenditure, and glucose metabolism, not only decreasing fat accumulation but also increasing muscle mass and function is an important area in obesity treatment [13, 14].

*Kaempferia parviflora* Wall ex. Baker* (K. parviflora)*, commonly known as black ginger, is herbaceous plant that belongs to the Zingiberaceae family [15]. Numerous studies have demonstrated the biological activities of* K. parviflora* including antiaging, anti-inflammatory, antiviral, and gastroprotective effects [15–17].* K. parviflora *contains abundant amounts of flavonoids and flavonoid glycosides. Among them, we found that the administration of 5,7-dimethoxyflavone (DMF), which is a major constituent of* K. parviflora*, attenuates obesity in high-fat-diet-induced C57BL/6J mice by downregulating adipogenesis [18, 19]. In addition,* K. parviflora* has been reported to improve physical fitness performance and muscular endurance in normal ddY mice [20]. Based on these considerations, we hypothesized that the ethanol extract of* K. parviflora* (KPE) might reduce obesity by preventing fat accumulation and improving muscle function in* ob/ob* mice. In this study, we investigated whether KPE attenuates fat accumulation by upregulating AMP-activated protein kinase (AMPK) and inhibits muscle atrophy by activating the phosphatidylinositol 3 kinase (PI3K)/Akt pathway in* ob/ob *mice.

## 2. Materials and Methods

### 2.1. Preparation of Standardized KPE

Dried rhizomes of* K. parviflora* were collected from Bangkok, Thailand. A specimen voucher was deposited in the Department of Biotechnology, Yonsei University (Seoul, Korea). The dried rhizomes of* K. parviflora *were ground and extracted with 95% ethanol for 3 h at 60°C. KPE was obtained by filtration and evaporation of the solvent with a yield of 8.9% (w/w). The amount of DMF in standardized KPE was measured by using the YL9100 HPLC system (Younglin, Anyang, Korea) with a Sunfire C18 column (150 mm × 4.6 mm id, 5 *μ*m; Waters, Milford, MA, USA). The standardized KPE contained 14.1% (w/w) DMF as a bioactive compound [19].

### 2.2. Animal Experiment

Five-week-old C57BL/6J (wild-type) and C57BL/6J* (ob/ob)* mice were purchased from SLC (Shizuoka, Japan) and housed under conditions of 55 ± 5% humidity, 12 h day/night cycle, and 25 ± 2°C. All mice were provided with a normal chow diet (Rodent Chow 38057; Purina Irradiated Lab, St. Louis, MO, USA) and water ad libitum throughout the experiment. After 3 weeks of acclimatization, the wild-type mice comprised the WT group (*n* = 7), and the* ob/ob* mice were divided into three groups (*n* = 7) in which the average body weight of each group was equal. Mice in the KPE 100 and KPE 200 groups were orally given KPE dissolved in saline at doses of 100 mg/kg/day and 200 mg/kg/day, respectively, for eight weeks. Mice in the WT and* ob/ob* groups were given an equal volume of saline to that provided in the KPE-treated groups. Body weight and food intake were measured twice a week. After the mice were sacrificed, gastrocnemius (GA) muscle, tibialis anterior muscle, soleus muscle (SOL), extensor digitorum longus (EDL) muscle, and the epididymal, subcutaneous, and perirenal adipose tissues were separated, measured, and stored in liquid nitrogen at −70. All the experimental protocols were reviewed and approved by the Institutional Animal Care and Use Committee (IACUC) of the Yonsei Laboratory Animal Research Center (Permit No.: IACUC-201607-470-04).

### 2.3. Microcomputed Tomography Imaging

Microcomputed tomography (Micro-CT) images of abdominal fat and hindlimb muscle were taken and analyzed with an animal positron emission tomography/CT/single photon emission tomography system (INVEON; Siemens, Washington, DC, USA) at the Center for Evaluation of Biomaterials (Pohang Technopark, Pohang, Korea).

### 2.4. Grip Strength Test

The grip strength of the mice was evaluated using a Chatillon force measurement system (Columbus Instrument, Columbus, OH, USA) equipped with a pull bar. Combined forelimb and hindlimb grip strength and forelimb grip strength were measured at the end of the oral administration period. The system has an electronic digital force gauge that determines the peak force. Each mouse was held by the tail until it released the pull bar. Five consecutive tests were performed on each mouse to obtain the peak value.

### 2.5. Treadmill Test

An animal treadmill (LE8710MTS, Panlab, Barcelona, Spain) was used to measure the running endurance of the mice. The mice ran on the treadmill at a speed of 12 m/min on 0° incline to become acclimated to the test in advance. In the actual test, the mice ran at the speed of 12 m/min on 0° incline followed by an increase of 3 m/min every 20 min thereafter. After 60 min, the incline was increased by 5° every 20 min. The shock grid was set to deliver 0.2 mA of electricity which did not physically injure the animals. The time at which the mice were unable to run after 10 s of electric shock was defined as exhaustion.

### 2.6. Histological Analysis

Epididymal fat tissues and GA tissues fixed with 10% formalin solution were embedded in paraffin and stained with hematoxylin and eosin (H&E). The stained tissues were observed under an inverted microscope equipped with twin charge-coupled device cameras (Eclipse TE2000U, Nikon, Tokyo, Japan). The adipocyte size and the cross-sectional area of the muscle fiber were quantified using ImageJ software (version 1.47; National Institutes of Health, Bethesda, MD, USA), represented as relative values to the adipocyte size and muscle fiber of WT mice.

### 2.7. Western Blot Assay

Homogenized epididymal fat tissues and GA tissues were analyzed by Western blot assay according to the previous study [21]. The primary antibodies against phospho-AMPK (p-AMPK), AMPK, phospho-acetyl-CoA carboxylase (p-ACC), ACC, peroxisome proliferator-activated receptor gamma (PPAR*γ*), CCAAT/enhancer-binding protein alpha (C/EBP*α*), sterol regulatory element binding protein 1c (SREBP-1c), PPAR*γ* coactivator 1-alpha (PGC-1*α*), uncoupling protein 2 (UCP2), UCP3, phospho-PI3K (p-PI3K), PI3K, phospho-Akt (p-Akt), Akt, phospho-mammalian target of rapamycin (p-mTOR), mTOR, phospho-70-kDa ribosomal protein S6 kinase (p-p70S6K), p70S6K, phospho-eukaryotic initiation factor 4E binding protein 1 (p-4EBP1), 4EBP1, phospho-forkhead box O3a (p-FoxO3a), FoxO3a, and *α*-tubulin were purchased from Cell Signaling Technology (Beverly, MA, USA) and used in a 1 : 1000 dilution. Horseradish peroxidase-conjugated secondary antibodies (1 : 2500 dilution; Bethyl Laboratories, Inc., Montgomery, TX, USA) were used to visualize the proteins on the membrane. The proteins were detected using an enhanced chemiluminescence detection solution (Amersham Biosciences, Little Chalfont, UK) and visualized with the G:BOX image analysis system (Syngene, Cambridge, UK).

### 2.8. Reverse Transcription-Polymerase Chain Reaction (RT-PCR)

Homogenized epididymal fat tissues, GA tissues, and SOL tissues were analyzed by RT-PCR according to the previous study [19]. Total RNA was extracted using RNAiso Plus (Takara, Kusatsu, Japan). The cDNA was synthesized using Reverse Transcriptase Premix (Elpis Biotech, Daejeon, Korea) and amplified using HiPi PCR PreMix (Elpis Biotech) and primer pairs (Table 1) in a Gene Amp PCR System 2700 (Applied Biosystems, Foster City, CA, USA). PCR products were separated by gel electrophoresis and detected by G:BOX Chemi XL (Syngene).

### 2.9. Statistical Analysis

All experiments were conducted in triplicate. Data are presented as the mean ± standard deviation. SPSS version 23.0 (SPSS Inc., Chicago, IL, USA) was used to perform statistical analysis using one-way analysis of variance (ANOVA). Differences between means were compared using Duncan's test. ^*∗*^*p* < 0.05, ^*∗∗*^*p* < 0.01, and ^##^*p* < 0.01 were considered statistically significant.

## 3. Results

### 3.1. KPE Reduces Body Weight without Change in Food Intake

**Figure 1:**
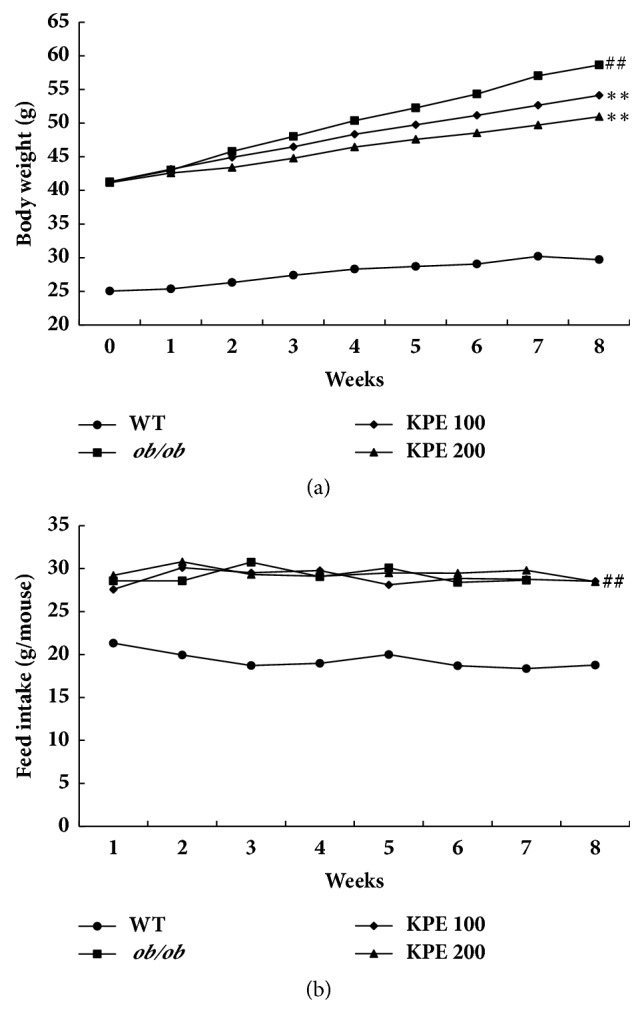
Effects of KPE on body weight and food intake. (a) Body weights. (b) Food intake. ^##^*p* < 0.01 compared to saline-treated WT group; ^*∗∗*^*p* < 0.01 compared to saline-treated* ob/ob *group.

At the end of the oral administration period, the* ob/ob* group had nearly twice the body weight of the WT group. KPE effectively reduced the body weights by 7.8% and 13.1% in the KPE 100 and KPE 200 groups, respectively (Figure 1(a)). KPE dose-dependently caused a 26.4% and 43.7% reduction in body weight gain in the KPE 100 and KPE 200 groups, respectively. During the oral administration period, the food intake was remarkably higher in the* ob/ob *mice than in the WT mice but did not differ among the* ob/ob* and KPE-treated groups (Figure 1(b)). These data show that KPE effectively reduces body weight* in vivo* by regulating metabolism without a reduction in appetite.

### 3.2. KPE Decreases Fat Size, Volume, and Mass

**Figure 2:**
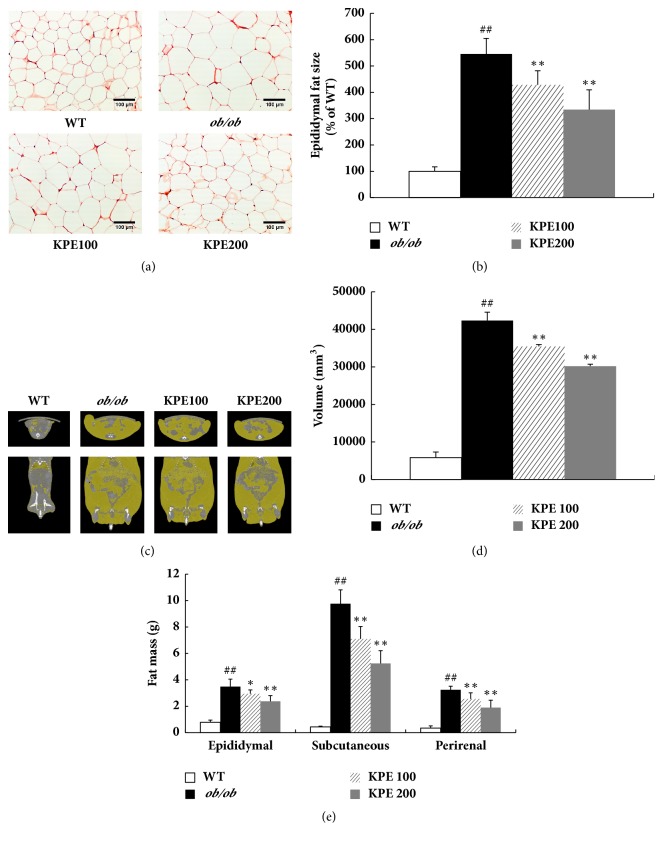
Effects of KPE on adipocyte size, abdominal fat volume, and fat tissue weights. (a) Representative H&E stain of epididymal fat tissue (magnification, ×200). (b) Epididymal fat size. (c) Microcomputed tomography images of abdominal fat. (d) Abdominal fat volume. (e) Weights of epididymal, subcutaneous, and perirenal fat. ^##^*p* < 0.01 compared to saline-treated WT group; ^*∗*^*p* < 0.05 and ^*∗∗*^*p* < 0.01 compared to saline-treated* ob/ob *group.

KPE greatly decreased adipocyte size in a dose-dependent manner (Figure 2(a)). In histological analysis, the adipocyte size of the* ob/ob* group was five times larger than that of the WT group. However, the KPE 100 and KPE 200 groups showed 21.6% and 38.7% decreases in adipocyte size, respectively, compared with the WT group (Figure 2(b)). To observe the effect of KPE on fat volume, we used micro-CT analysis (Figure 2(c)) and quantified the fat volume (Figure 2(d)). The results showed that the* ob/ob* group exhibited 7.23 times more fat volume than the WT group, while KPE significantly lowered fat volume in a dose-dependent manner (Figure 2(d)). The* ob/ob* group had significantly higher adipose tissue weights than the WT group; however, KPE dose-dependently decreased the adipose tissue weights (Figure 2(e)). In the KPE 100 group, the epididymal, subcutaneous, and perirenal adipose depot were decreased by 15.2%, 27.3%, and 20.9%, respectively. In the KPE 200 group, the three adipose depot weights were decreased by 31.6%, 46.4%, and 41.4%, respectively. These results indicate that oral administration of KPE reduces adipocyte size and fat volume, leading to a significant reduction in fat accumulation* in vivo*.

### 3.3. KPE Downregulates Expression of Adipogenic Transcription Factors and Lipogenic Enzymes through AMPK Activation in Fat

**Figure 3:**
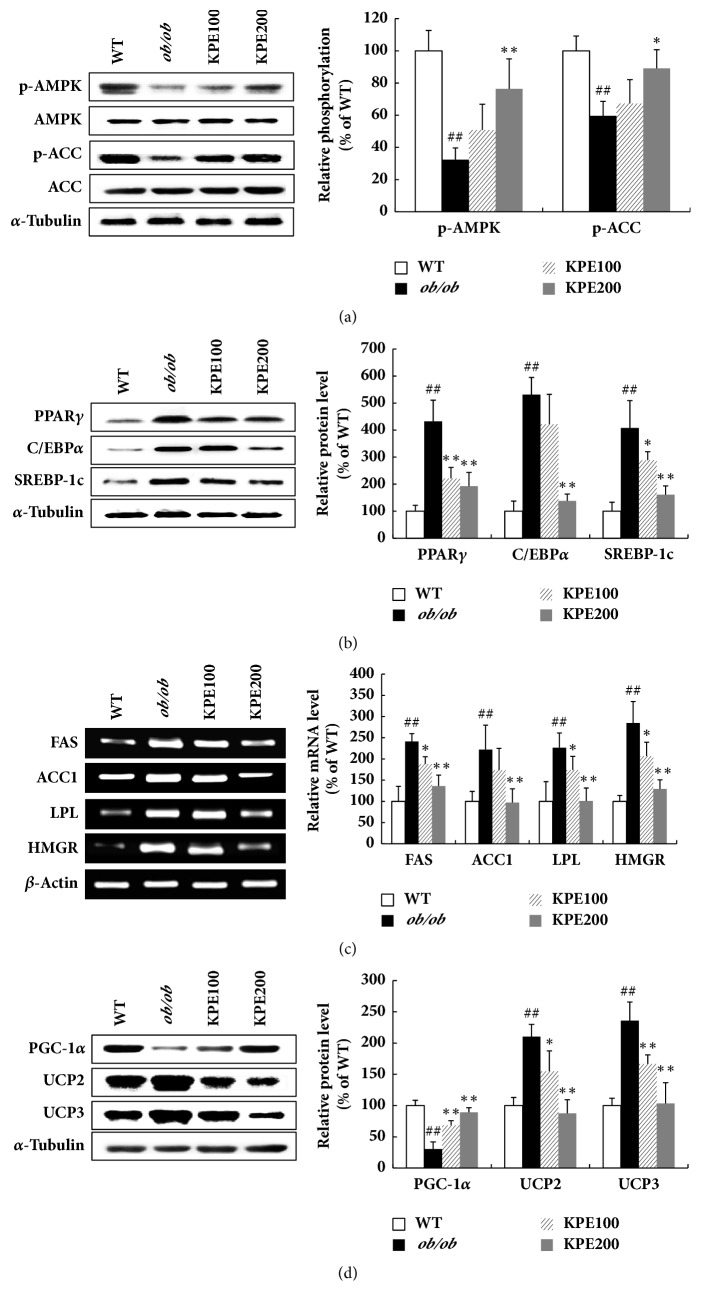
Effect of KPE on adiposity in epididymal fat. (a) Relative protein levels of p-AMPK, AMPK, p-ACC, and ACC. (b) Relative protein levels of PPAR*γ*, C/EBP*α*, and SREBP-1c. (c) Relative mRNA levels of FAS, ACC1, LPL, and HMGR. (d) Relative protein levels of PGC-1*α*, UCP2, and UCP3. ^##^*p* < 0.01 compared to saline-treated WT group; ^*∗*^*p* < 0.05 and ^*∗∗*^*p* < 0.01 compared to saline-treated* ob/ob *group.

Epididymal fat tissue is a white adipose tissue sensitive to obesity [22, 23]. Thus, we analyzed the molecular mechanism with epididymal fat tissue. In the epididymal fat tissue, KPE stimulated phosphorylation of AMPK and ACC, a downstream target of AMPK, while no change was observed in total protein expression of AMPK and ACC (Figure 3(a)). KPE reduced the protein expression levels of adipogenic transcription factors such as PPAR*γ*, C/EBP*α*, and SREBP-1c (Figure 3(b)). In addition, the mRNA levels of lipogenic enzymes such as lipoprotein lipase (LPL), ACC1, fatty acid synthase (FAS), and HMG-CoA reductase (HMGR) were downregulated by KPE in a dose-related manner (Figure 3(c)). The protein expression of PGC-1*α* was elevated, while the expressions of UCP2 and UCP3 were reduced in the KPE-treated groups (Figure 3(d)). These results demonstrate that KPE reduces fat accumulation and modulates energy metabolism by downregulating adipogenic transcription factors and lipogenic enzymes.

### 3.4. KPE Improves Endurance Exercise and Grip Strength

**Figure 4:**
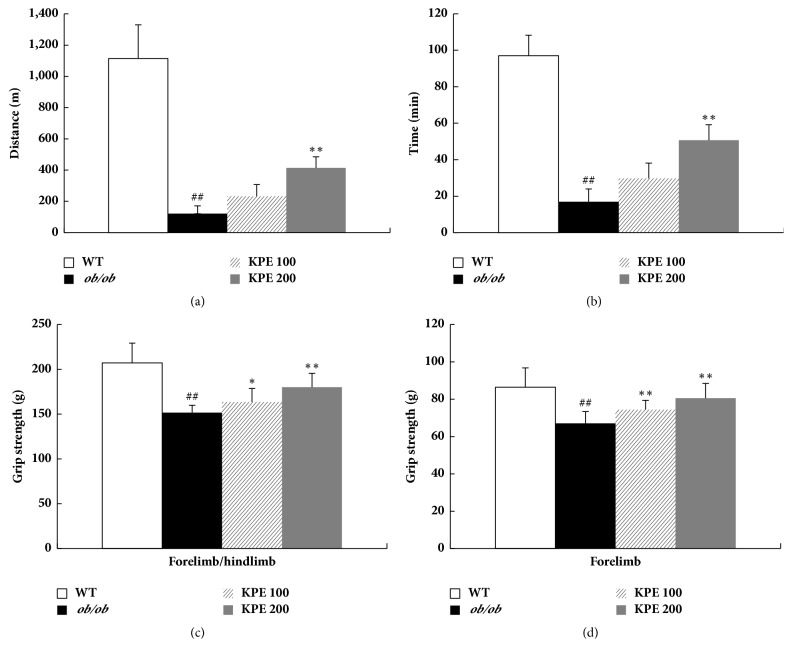
Effects of KPE on running endurance and grip strength. (a) Running distance. (b) Running time. (c) Grip strength of fore- and hindlimb. (d) Grip strength of forelimb. ^##^*p* < 0.01 compared to saline-treated WT group; ^*∗*^*p* < 0.05 and ^*∗∗*^*p* < 0.01 compared to saline-treated* ob/ob *group.

Previously, KPE has been reported to improve physical fitness performance in normal mice [20]. In this study, we used a treadmill and grip strength meter to observe whether KPE enhances muscle function in* ob/ob* mice. The* ob/ob* group showed markedly decreased running endurance on an accelerating treadmill compared with the WT group (Figures 4(a) and 4(b)). The KPE 100 and KPE 200 groups showed increased running distances by 1.9 and 3.4 times, respectively, compared with the* ob/ob* group (Figure 4(a)). The grip strengths of the KPE groups were also remarkably higher than those of the* ob/ob* group. Combined forelimb and hindlimb grip strengths in the KPE 100 and KPE 200 groups were shown to increase by 15.32 ± 13.68 g and 22.52 ± 16.88 g, respectively, compared with the* ob/ob* group (Figure 4(c)). The forelimb grip strengths in the KPE 100 and KPE 200 groups were increased by 8.19 ± 7.68 g and 12.09 ± 6.86 g, respectively (Figure 4(d)). These results suggest that the hindlimb strength might be increased by KPE treatment. Collectively, KPE stimulates skeletal muscle anabolism* in vivo*, leading to muscle hypertrophy, improved endurance exercise, and increased muscle strength.

### 3.5. KPE Increases Skeletal Muscle Fiber Size, Volume, and Mass

**Figure 5:**
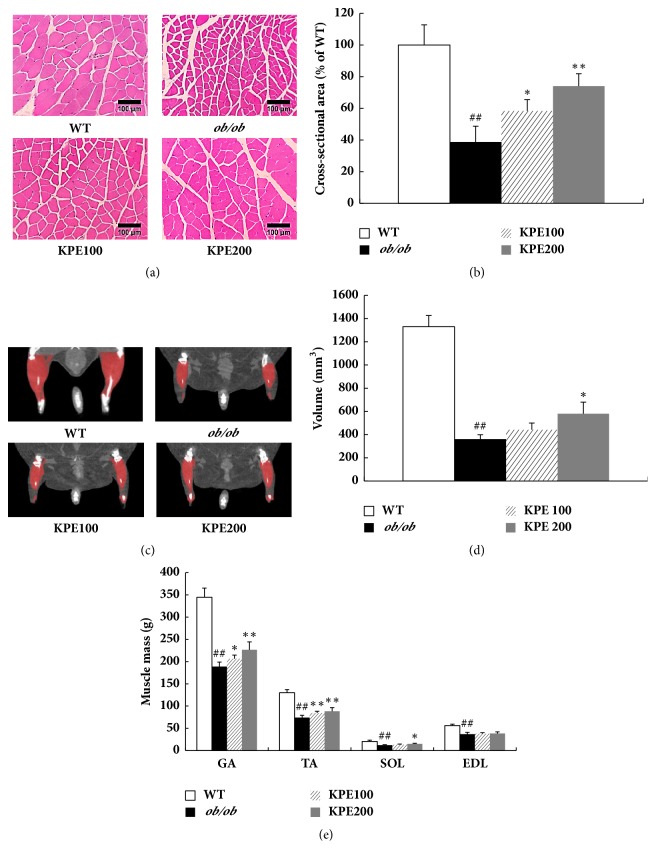
Effect of KPE on muscle fiber size, hindlimb muscle volume, and muscle weights. (a) Representative H&E stain of gastrocnemius muscle fibers (magnification, ×200). (b) Gastrocnemius muscle fiber cross-sectional area. (c) Microcomputed tomography images of hindlimb muscle. (d) Hindlimb muscle volume. (e) Weights of gastrocnemius (GA), tibialis anterior (TA), soleus (SOL), and extensor digitorum longus (EDL) muscles. ^##^*p* < 0.01 compared to saline-treated WT group; ^*∗*^*p* < 0.05 and ^*∗∗*^*p* < 0.01 compared to saline-treated* ob/ob *group.

In addition to adipose tissue, we examined the effect of KPE on skeletal muscle* in vivo*. Interestingly, the cross-sectional area of muscle fiber of the* ob/ob* group was only 38.7% that of the WT group (Figures 5(a) and 5(b)). However, KPE treatment greatly increased the cross-sectional area of muscle fiber in a dose-dependent manner. It also increased skeletal muscle volume in the KPE 100 and KPE 200 groups by 22.2% and 60.4%, respectively, compared with the* ob/ob* group (Figures 5(c) and 5(d)). The GA muscle mass was increased by 9.3% and 20.0%, the TA muscle mass was increased by 14.5% and 19.5%, and the SOL muscle mass was increased by 8.2% and 23.2% in the KPE 100 and KPE 200 groups, respectively (Figure 5(e)). A tendency for the weight of the EDL muscle to increase was also observed, but there was no statistically significant change. Collectively, KPE improves muscle atrophy by increasing muscle fiber size, muscle volume, and muscle mass in* ob/ob* mice.

### 3.6. KPE Stimulates PI3K/Akt Pathway in Skeletal Muscle

**Figure 6:**
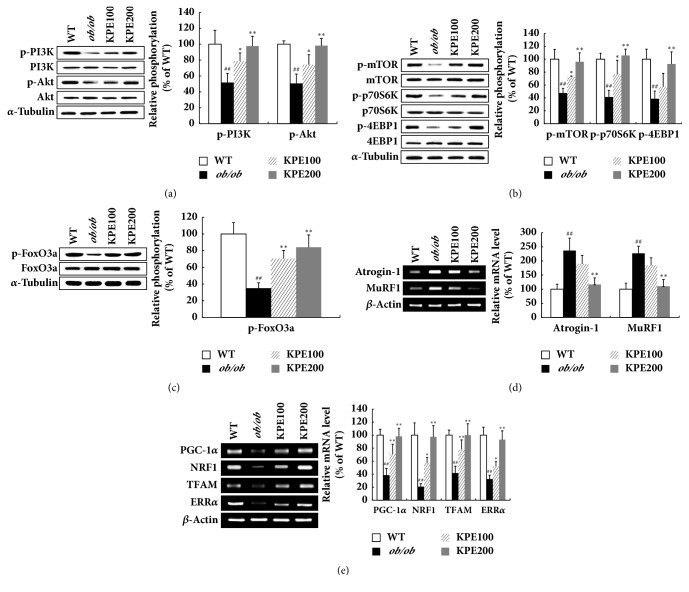
Effect of KPE on PI3K/Akt pathway in gastrocnemius muscle. (a) Relative phosphorylation of PI3K and Akt. (b) Relative phosphorylation of mTOR, p70S6K, and 4EBP1. (c) Relative phosphorylation of FoxO3a. (d) Relative mRNA levels of Atrogin-1 and MuRF1. (e) Relative mRNA levels of PGC-1*α*, NRF1, TFAM, and ERR*α*. ^##^*p* < 0.01 compared to saline-treated WT group; ^*∗*^*p* < 0.05 and ^*∗∗*^*p* < 0.01 compared to saline-treated* ob/ob *group.

Activation of PI3K/Akt pathway is a key regulator in protein anabolism [24]. As KPE greatly increased skeletal muscle growth* in vivo*, we investigated its effect on PI3K/Akt in muscle. In the* ob/ob* group, the expression levels of p-PI3K and p-Akt were suppressed by up to 46% compared with the WT group (Figure 6(a)). However, KPE markedly upregulated the expression of the PI3K/Akt pathway, followed by the mTOR pathway (Figures 6(a) and 6(b)). In addition, the expression of p-FoxO3a, the inactive form of FoxO3a, was significantly increased by KPE (Figure 6(c)). In contrast, atrogin-1 and muscle ring-finger protein-1 (MuRF1), the target genes of FoxO3a, were downregulated by KPE (Figure 6(d)). Meanwhile, KPE elevated the mRNA levels of mitochondrial biogenesis-related biomarkers in soleus muscle (Figure 6(e)). From these results, KPE might stimulate muscle growth by activating the PI3K/Akt pathway and enhance exercise endurance by increasing mitochondrial biogenesis.

## 4. Discussion

There have been many attempts to identify natural products that counteract obesity [25]. Among numerous natural antiobesity agents, only a few have been reported to have muscle hypertrophic effect [21, 26]. In the present study, we provide evidence of the dual function of KPE on obesity and muscle atrophy in* ob/ob* mice.

Obesity is characterized by increased body weight caused by abnormal adipose tissue growth [2]. During the past decade, AMPK has been targeted as a therapeutic approach for obesity treatment since it plays a pivotal role in energy metabolism [6, 7]. Activation of AMPK inhibits fatty acid synthesis via the inactivation of lipogenic enzymes such as ACC1, HMGR, and FAS [7]. Moreover, AMPK activation inhibits adipogenesis by downregulating PPAR*γ*, C/EBP*α*, and SREBP-1c, which are highly expressed during adipocyte differentiation and regulate the expression of multiple adipogenic proteins such as LPL, glucose transport-4, and adipocyte fatty acid-binding protein [27–29]. In contrast, AMPK facilitates fatty acid oxidation by upregulating lipolytic and thermogenic proteins, such as PGC-1*α* and UCPs [7, 29].

In this study, KPE effectively decreased the epididymal, subcutaneous, and perirenal fat tissues (Figure 2(e)). Epididymal fat tissue, a white adipose tissue sensitive to obesity [22, 23], was used to investigate molecular mechanism. Oral administration of KPE activated the phosphorylation of AMPK in epididymal fat tissue (Figure 3(a)). Consequently, AMPK activation led to the decreased expression of adipogenic transcription factors including PPAR*γ*, C/EBP*α*, and SREBP-1c and lipogenic enzymes including ACC1, HMGR, FAS, and LPL (Figure 3(c)). These results are consistent with our previous finding that DMF attenuates obesity by inhibiting adipogenesis and lipogenesis [19]. Since the standardized KPE used in this research contained 14.11% DMF as a main bioactive compound, its antiobesity effect through stimulating lipolysis and blocking a process of adipogenesis can be attributed to DMF. Meanwhile, the mRNA and protein levels of UCPs are known to be more upregulated in* ob/ob* mice than in WT mice [30, 31]. Interestingly, UCPs were downregulated in the KPE-treated groups compared with the* ob/ob* group (Figure 3(d)). This is possibly due to fat reduction by KPE, since decreased fat can reduce the need for heat generation and fatty acid oxidation. Additionally, the active components of KPE, 5,7,4′-trimethoxyflavone and 3,5,7,3′,4′-pentamethoxyflavone, were reported to prevent adipocyte hypertrophy by activating lipolytic enzymes in mature 3T3-L1 adipocytes [18, 32]. Thus, in addition to DMF, the two methoxyflavones in KPE might have contributed to the fat reduction in* ob/ob* mice. These results show that KPE effectively reduces obesity and downregulates adiposity via the activation of the AMPK signal cascade in fat tissue.

The PI3K/Akt pathway is a major pathway involved in muscle protein anabolism which regulates the mTOR pathway [33]. The activation of mTOR is another critical event in skeletal muscle growth because it promotes certain mechanisms, such as protein formation and mitochondrial biogenesis by increasing expression of insulin-like growth factor-1 (IGF-1) and PGC-1*α*, respectively [21, 33]. Furthermore, Akt phosphorylates FoxO3a protein, which is a key regulatory factor in protein degradation, leading to its sequestration in the cytoplasm away from its target genes [24]. Out of the four muscles constituting the hindlimb, GA muscle occupies the highest proportion [34]. Thus, molecular mechanism related to muscle growth was investigated with GA muscle. In this study, KPE promoted the PI3K/Akt pathway activation (Figure 6(a)) in GA muscle and consequently led to muscle growth (Figure 5) by activating mTOR signaling and repressing FoxO3a (Figures 6(b) and 6(c)). Because an increase in Akt activity in muscle reduces adiposity as a secondary consequence of muscle growth, increased Akt activity by KPE might have contributed to fat reduction [35]. Collectively, KPE both stimulates skeletal muscle anabolism and suppresses adiposity in* ob/ob* mice.

An increase in type II muscle mass leads to the enhancement of muscle strengths [35]. KPE significantly increased the cross-sectional area of muscle fibers which led to the increased weights of type II muscles, GA and TA muscles (Figure 5(e)). The increase in GA and TA muscle weights resulted in an increase in grip strengths (Figure 4(c)). In contrast, an increase in type I muscle mass causes an improvement of endurance exercise [36]. KPE increased the soleus muscle weight and greatly enhanced running endurance. Furthermore, KPE upregulated the mRNA level of mitochondrial biogenesis-related biomarkers, such as PGC-1*α*, nuclear respiratory factor 1 (NRF1), mitochondrial transcription factor A (TFAM), and estrogen-related receptor *α* (ERR*α*) in soleus muscle (Figure 6(e)). Previous studies have reported that KPE increased the amount of mitochondrial DNA and glycogen* in vivo* and promotes energy production by upregulating ATP production and AMPK in C2C12 myocytes [20, 37]. Therefore, KPE may enhance running endurance by improving the function and quality of type I fibers, not just through its hypertrophy in size. However, further investigation is required to clarify which molecular mechanism of KPE has a direct effect on endurance exercise in obese mice.

## 5. Conclusions

In the present study, we found that KPE significantly reduced body fat without a change in appetite, increased skeletal muscle mass, and improved muscle function in* ob/ob* mice. These results are associated with the increased activation of AMPK in fat and the PI3K/Akt pathway in muscle. Thus, KPE has two major effects on body composition: the abilities to inhibit fat anabolism and stimulate skeletal muscle anabolism. These preclinical data recommend further investigation in humans. Collectively, the results strongly suggest that KPE could be used as a functional food material to attenuate obesity and increase muscle mass and function.

## Figures and Tables

**Table 1:** Primer sequences used in RT-PCR analysis.

Origin	Gene	Direction	Sequence (5′-3′)
Mouse	FAS	Forward	CTGCGGAAACTTCAGGAAATG
		Reverse	GGTTCGGAATGCTATCCAGG
	ACC1	Forward	AGGAGGACCGCATTTATCGAC
		Reverse	TGACCGTGGGCACAAAGTT
	LPL	Forward	TTGCGCCTCCTGCTCAACCC
		Reverse	CCCCTCCTCGGAAGGCGGTC
	HMGR	Forward	CTGCGGAAACTTCAGGAAATG
		Reverse	GGTTCGGAATGCTATCCAGG
	PGC-1*α*	Forward	GTCCTTCCTCCATGCCTGAC
		Reverse	GACTGCGGTTGTGTATGGGA
	NRF1	Forward	CTTCATGGAGGAGCACGGAG
		Reverse	ATGAGGCCGTTTCCGTTTCT
	TFAM	Forward	GAGCGTGCTAAAAGCACTGG
		Reverse	CCACAGGGCTGCAATTTTCC
	ERR*α*	Forward	AGTGTGAGATCACCAAGCGG
		Reverse	GGCGTACAGCTTCTCAGGTT
	*β*-Actin	Forward	CCAGCCGAGCCACATCGCTC
		Reverse	TGACCTTGGCCAGGGGTGCT

## Data Availability

All relevant data supporting the findings of this study are within the paper.
